# Development of a Simvastatin‐Loaded Copolymer Acid‐Sensitive Nanocarrier and Its Experimental Use in the Treatment of Keloids

**DOI:** 10.1111/jocd.16573

**Published:** 2024-09-23

**Authors:** Bin‐yu Zhuang, Fang‐chi Hu, Xuan Gao, Qi Leng, Ying Zhang, Yan You

**Affiliations:** ^1^ Department of Dermatology Fourth Affiliated Hospital of Harbin Medical University Harbin China; ^2^ Department of Orthopaedics Harbin First Hospital Harbin China; ^3^ Harbin Medical Sciences University Harbin China; ^4^ Department of Geriatrics The First Affiliated Hospital of Zhejiang University School of Medicine Hangzhou China; ^5^ Department of Dermatology Huizhou Institute of Skin Disease Prevention and Control Huizhou China

**Keywords:** complex nanocontrolled delivery system, fibroblasts, keloid, PEG, PH, PLGA, simvastatin

## Abstract

**Objective:**

The lipid‐lowering simvastatin (SIM) has been shown to be an effective inhibitor of keloid proliferation. However, due to its low water solubility and short half‐life, simvastatin aggregates to the liver and does not reach the skin lesions after oral administration, which restricts its widespread clinical use. The development of nanomedicine provides the possibility for us to break through this bottleneck problem clinically. The objective of this study was to investigate the feasibility of using complex nanocontrolled delivery system (CNDS), simvastatin‐loaded polyethylene glycol‐poly lactic‐co‐glycolic acid (PEG‐PLGA), in the treatment of keloids.

**Methods:**

In the in vitro study, the release of simvastatin in fibroblasts by CNDS@Simvastatin and its effect on inhibition of the proliferation of fibroblasts, Col Ι, and CTGF by the slow release of simvastatin were assessed. The efficacy of CNDS@Simvastatin in improving keloids and the biocompatibility and safety of CNDS@Simvastatin were examined in vivo by establishing a murine ear keloid model.

**Results:**

CNDS@Simvastatin showed sustained and uniform inhibition of the proliferation of fibroblasts, Col Ι, and CTGF via the gradual release of simvastatin over 72 h. It was efficient in the treatment of the murine ear keloid with no observable toxic side effects on various organs.

**Conclusion:**

Simvastatin‐loaded copolymer acid‐sensitive nanocarriers, CNDS@Simvastatin nanospheres, were successfully developed in this study, and these were characterized by favorable physicochemical properties and biocompatibility.

## 1 Introduction

Keloid, a fibroproliferative reaction to wound healing in which scar tissue grows beyond the original wound margin following skin injury or irritation, belongs to a special category of pathological scars [1, 2]. Characterized by masses that protrude over the normal skin surface beyond the site of the original injury, keloids show persistent growth, a hard texture, poor elasticity, and a growth pattern similar to tumors. They are often accompanied by itching or pain and can seriously impact the physical and psychological well‐being of patients [3, 4].

At present, the therapeutic management of keloids mainly includes intra‐scar drug injection, and cryotherapy, photoelectric therapy, surgical resection, radiotherapy, etc. Despite the array of treatment modalities, treatment outcomes remain unsatisfactory, and the recurrence rate is high. Surgical resection alone has a recurrence rate of 45%–100% [5]. Consequently, surgery has been combined with intra‐scar drug injection [6], and liquid nitrogen cryotherapy [7], postoperative radiotherapy, and standalone radiotherapy. This underscores the need for a treatment method for keloids that is safe, effective, and has a low recurrence rate.

Keloids are characterized histologically by an imbalance between proliferation and apoptosis of fibroblasts, increased extracellular matrix (ECM), and intertwined and ill‐defined collagenous fibers with thick hyperplasia [8]. Transforming growth factor beta 1 (TGF‐β1) binds to fibroblast surface receptors and forms heterotrimers, which transduce connective tissue growth factor (CTGF) into the nucleus by activating the Smad2/3/4 protein in the cytoplasm and nucleus. CTGF, in turn, induces the transformation of fibroblasts into myofibroblasts, ultimately stimulating increased dermal ECM secretion, thereby increasing the synthesis of collagen type I (Col I) and collagen type III (Col III), as well as promoting collagen fiber contraction [9]. This is the TGF‐β1/Smad signaling pathway within keloid fibroblasts. Therefore, exploring the effects of various factors on the proliferation of fibroblasts, CTGF, and ECM, regardless of the pathological findings of keloids or their TGF‐β1/Smad signaling pathways, assumes significance.

Simvastatin, a pharmaceutical agent that is clinically prescribed for lowering blood lipids, has been shown in early experiments to be an effective inhibitor of TGF‐β1‐induced proliferation of type I collagen and CTGF in keloid fibroblasts [1]. However, due to its low water solubility and short half‐life, simvastatin does not achieve sufficient accumulation in the liver for subsequent delivery to the skin lesions after oral administration. Furthermore, repeated intralesional injections heighten the risk of skin rhabdomyolysis. All these factors have resulted in its low utilization rate. Therefore, the clinical use of simvastatin in the treatment of keloids remains limited.

The advent of nanomedicine presents a promising possibility for overcoming these challenges and achieving a breakthrough in clinical practice. Leveraging materials with good biocompatibility can ensure safety and mitigate adverse effects. To prolong the circulation time of the drug in the blood, a method using slow‐release diblock nanopolymer micelles composed of polyethylene glycol‐poly lactic‐co‐glycolic acid (PEG‐PLGA) was developed to encapsulate simvastatin within the human body with targeted injection into keloid lesions. The PEG component situated in the outer part of the micelles facilitates movement in the blood and extracellular matrix, thereby prolonging the circulation time of the micelles [10]. Inside the micelles, simvastatin is encapsulated within the PLGA component to facilitate controlled, slow release of the drug [11, 12].

**FIGURE 1:**
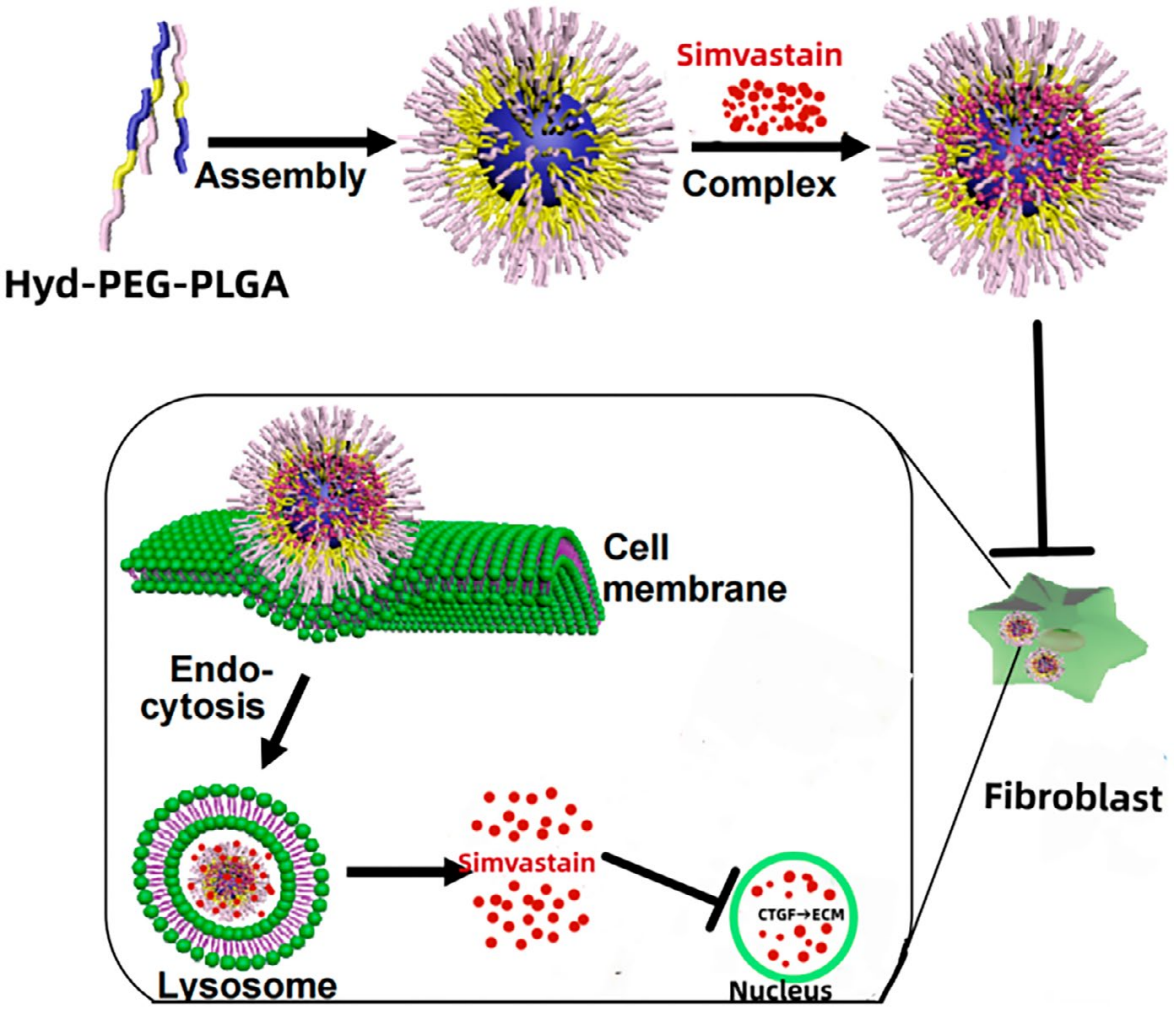
Schematic diagram of drug release after CNDS@Simvastatin enters fibroblasts.

However, following the intralesional injection of PEG‐PLGA‐loaded simvastatin nanomicelles, it was observed that simvastatin was released either slowly or prematurely from the micelles, resulting in insufficient inhibition of fibroblasts and a poor drug utilization rate. Consequently, the design was remodified, resulting in simvastatin copolymer‐loaded acid‐sensitive nanomicelles, denoted as CNDS@Simvastatin. As shown in Figure 1, PEG‐PLGA microsphere pores were added to the pH‐sensitive chemical bond hyd (CNDS) to co‐encapsulate Simvastatin, thus yielding CNDS@Simvastatin nanospheres.

The anticipated outcome was a controlled release profile wherein simvastatin remained largely retained within the micelles in a pH 7.4 extracellular environment, with minimal release occurring. Upon cellular uptake by fibroblasts and subsequent exposure to the acidic milieu of lysosomes (pH 5.0–6.5), hyd dissociation would ensue, thereby enabling the slow release of the PLGA‐encapsulated simvastatin. The ultimate goal was to achieve the treatment of keloids by enhancing the drug utilization rate through sustained‐release Simvastatin and uniform inhibition of fibroblasts, CTGF, and ECM proliferation.

The objectives of our experiment were to develop CNDS@Simvastatin and evaluate its characterization and performance, as well as verify whether CNDS@Simvastatin could release drugs in fibroblasts slowly and inhibit the proliferation of fibroblasts, CTGF, and ECM at the cellular level in vitro. We additionally explored the efficacy of CNDS@Simvastatin in the treatment of keloids in a murine ear keloid model and evaluated its toxicity and side effects on vital organs in mice in vivo, along with its biocompatibility.

## 2 Materials and Methods

### 2.1 Preparation and Characterization of CNDS@Simvastatin

#### 2.1.1 Synthesis of the PEG‐PLGA‐Hyd Copolymer Containing Simvastatin

Synthesis of the PEG‐PLGA copolymer: Carboxyl activation in PLGA was followed by the addition of PEG with predetermined relative molecular masses in a molar ratio of 1:1. This mixture was placed in a 100‐mL three‐port flask, and methanol was instilled proportionally under nitrogen protection at normal pressure. The resulting precipitate was collected through multiple centrifugation steps, yielding 910 mg of PEG‐PLGA (390 mg of PEG and 520 mg of PLGA) copolymer after vacuum drying for 3 days.

The pH‐sensitive chemical bond Hyd was introduced into the PEG‐PLGA copolymer through an anhydride reaction. A quantity of 500 mg of PEG‐PLGA, 50 mg of N‐hydroxysuccinimide (NHS), 270 mg of diluted hydrochloric acid (HCL) in 30 mL of deionized water, and 295 mg of tert‐butyl hydrazine formate in 10 mL of deionized water were combined. Additionally, 200 mg of simvastatin and 163 mg of CNDS were dissolved in 30 mL of methanol using acetic acid catalysis. The resulting mixture was subjected to dialysis for 3 days, followed by freeze‐drying for another 3 days.

#### 2.1.2 Determination of the Properties of CNDS@Simvastatin

Transmission electron microscopy (Shenzhen Speed Instrument Co., Shenzhen, China) was used to determine the morphology of CNDS@Simvastatin. The particle size and particle morphology of the sample were ascertained by subjecting the powder to TEM analysis. Subsequently, the sample was diluted with CNDS@Simvastatin and negatively stained with phosphotungstic acid. The solvent was evaporated to dryness and observed under TEM.

#### 2.1.3 Determination of Particle Size and Zeta Potential of CNDS and CNDS@Simvastatin Using Dynamic Light Scattering

The sample was dissolved in ultrapure water and filtered through a 0.22 μM microporous filter membrane. The instrument, cuvette, and potential testing device were sequentially rinsed with ultrapure water and the sample solution. The samples were added, and their particle size and zeta potential were determined using dynamic light scattering (DLS) (Shenzhen Speed Instrument Co., Shenzhen, China).

#### 2.1.4 Determination of Entrapment Efficiency and Drug Loading of CNDS@Simvastatin

The absorption values of Lambda800 samples were detected using a UV–visible photometer (Thermo Fisher Scientific China Ltd., Shanghai, China) at 238 nm and calculated using the standard curve equation. Subsequently, encapsulation efficiency was calculated as per the following formula:

$$\mathrm{EN}\%=\left(1-C_{f}/C_{t}\right)\times 100\%$$

where *C*_f_ represents the amount of free drug and *C*_t_ represents the total amount of drug in nanoparticles.

Drug loading was calculated as per the following formula:

LC=W2/W1×100%

where W1 denotes the weight of the lyophilized nanoparticle sample; W2 is the drug content detected according to the drug test method.

An amount of 150 mg of CNDS@Simvastatin sample was weighed and dissolved into a volumetric flask. Dimethylsulfoxide was added for dissolution. Using the aforementioned formulas. The drug content detected in the obtained product was 4.9 mg.

#### 2.1.5 Analysis of the Performance of CNDS@Simvastatin

##### 2.1.5.1 Drug Release Behavior of CNDS@Simvastatin

In order to investigate the in vitro drug release behavior of CNDS@Simvastatin, two different release media, pH 7.4 PBS and pH 5.0 PBS, were selected. Samples were taken at various time points (0, 0.5, 1, 2, 4, 8, 12, 24, 48, and 72 h) and filtered. Absorbance was measured using UV spectrophotometry, and drug release profiles were plotted.

##### 2.1.5.2 Evaluation of the Stability of CNDS@Simvastatin

The particle size of the nanosystem was determined using DLS, and the stability of CNDS and CNDS@Simvastatin in normal saline (NS) solution was evaluated. After filtering with a 0.22 μM microporous filter membrane, the cuvette, and potential testing device were sequentially rinsed with the sample solution. DLS measurements of the particle size were obtained on day 0, day 2, day 4, day 6, and day 8 to determine the stability of the formulation.

##### 2.1.5.3 In Vitro Screening of Drug Cell Proliferation Assay (MTT Assay)

For the MTT assay, cells were seeded into 96‐well plates, at a density of 5 × 10^3^ cells/well. Once the cell confluence reached approximately 90%, varying concentrations of CNDS, simvastatin, and CNDS@Simvastatin were added to the 96‐well plates, and incubation was continued for 24 h. Later, the drugs and culture medium were removed from the 96‐well plate, and 200 μL of fresh culture medium and 20 μL of a 5 mg/mL MTT solution were added to each well. The plate was then incubated for 4 h at 37°C. Absorbance readings were obtained using a microplate reader. Computational mapping was used to examine the effects of different concentrations of CNDS, Simvastatin, and CNDS@Simvastatin on cell viability.

##### 2.1.5.4 Evaluation of Cellular Uptake

Cellular uptake was assessed by encapsulating CNDS and CNDS@Simvastatin with the green fluorescent dye FAM using emulsification‐solvent evaporation, which yielded CNDS@FAM and CNDS@SimvastatinFAM, respectively. Human umbilical vein endothelial cells (HUVEC) were seeded into confocal culture dishes and co‐cultured with saline, CNDS@FAM, and CNDS@SimvastatinFAM for 24 h. Qualitative analysis was conducted with Confocal Laser Scanning Microscopy (Chengdu Century Meiyang Technology Co., Chengdu, China) by staining the cells with Hoechst 33342 for 15 min at room temperature.

### 2.2 Experimental Study of Simvastatin‐Loaded Copolymer Acid‐Sensitive Nanocarriers on Keloid Fibroblasts In Vitro

#### 2.2.1 Experimental Grouping

Control group (control group): fibroblasts.

Experimental group: (1) blank vector group (CNDS group); (2) simvastatin group (Simvastatin group); and (3) acid‐sensitive nanomaterial simvastatin group (CNDS@Simvastatin group).

Cultures were performed separately with fibroblasts for each experimental group.

#### 2.2.2 Proliferating Cell Nuclear Antigen Antibody Staining Using Confocal Microscopy to Observe the Inhibition of Fibroblast Proliferation in Each Group

Cells were permeabilized with 0.1% TritonX‐100 for approximately 15 min at room temperature. Fibroblasts were localized in paraformaldehyde and washed with phosphate buffer for 3 min. The blocking solution was removed, and 40 μL of primary antibody was added to the cell plate to cover the cell tissue, which was then stored in the dark and photographed.

#### 2.2.3 Release of CNDS@Simvastatin in Fibroblasts

Cell culture incubation: The material and human skin fibroblasts (HSFs) were co‐cultured for 24 h. Cy7‐labeled nanospheres and DAPI‐labeled nuclei were used. Immunofluorescence confocal imaging was conducted to detect the uptake of the material by cells and then photographed.

#### 2.2.4 Quantitative Polymerase Chain Reaction Assay

The quantitative polymerase chain reaction (Q‐PCR) assay involved RNA extraction followed by RNA purity determination and RNA quantification. Reverse transcription experiments were subsequently performed.

#### 2.2.5 Western Blot Analysis

Cell protein extraction: The cell culture supernatant was aspirated, and a certain amount of radio immunoprecipitation assay (RIPA) lysate was added. The cells were then lysed by shaking on ice for 15 min. Following this, gel preparation, electrophoresis, transfer, blocking, incubation with the primary antibody, and subsequent incubation with the secondary antibody were performed.

#### 2.2.6 Cellular Cell Counting Kit‐8 Assay

Cells were seeded onto plates, and 0.25% trypsin [containing Ethylene Diamine Tetraacetic Acid (EDTA)] was used to digest the cells for approximately 2 min at 37°C. The cells were then collected and centrifuged at 1000 rpm for 4 min. A 20‐μL cell suspension was taken, and 20 μL of trypan blue solution was added. Subsequently, 100 μL of fresh medium containing 0.5% Fetal Bovine Serum (FBS) was added into each well of a 96‐well plate, followed by the addition of 10 μL of cellular cell counting kit‐8 (CCK8). The plate was then incubated for 2 h at 37°C. Data analysis was done using Prism 5.

#### 2.2.7 Cell Apoptosis Experiment

After cells were seeded onto plates, 0.25% trypsin (containing EDTA) was added to digest the cells for about 1 min at 37°C, and the cells were collected. Annexin V‐Fluorescein isothiocyanate (V‐FITC) and propidium iodide (PI) were utilized for apoptosis detection by flow cytometry. Annexin V‐FITC emits green fluorescence, while PI emits red fluorescence. The green fluorescence excitation wavelength of FITC is 488 nm.

### 2.3 In Vivo Experimental Study of the Effect of Simvastatin‐Loaded Copolymer Acid‐Sensitive Nanocarriers on Keloids

#### 2.3.1 Source of Experimental Animals and Feeding Protocol

Sixteen Institute of Cancer Research (ICR) mice, aged 7–8 weeks and weighing 20 ± 5 g with no specific disease, were purchased from Kexing Biopharmaceutical Co., Ltd. (SYXK (Lu) 21–0033). All mice were housed under controlled conditions at 25°C and 40% humidity, with a 12‐h light–dark cycle and free access to food and water.

Development of the animal model: Procedures for handling animals were as per the *Guidelines for the Care and Use of Laboratory Animals* [13]: Six‐week‐old female ICR‐grade mice without any ear skin disorders were used to develop a murine keloid ear model. Following general anesthesia, wounds were created on the exposed cartilage ventral to each ear with a 2‐mm dermal biopsy punch. Contralateral ears from each group served as controls between groups.

Scar formation was observed at 15 days after surgery, and mice with poor scar formation and postoperative infections were excluded from the experimental group. The remaining mice were randomly divided into four groups: the control group (*n* = 4), in which scar formation was induced without injection of any drug; the CNDS group (*n* = 4), in which blank carriers of the unembedded drug were injected locally into the scar; the simvastatin group (*n* = 4), in which simvastatin was injected locally into the scar; and the CNDS@Simvastatin group (*n* = 4), in which polymer nanocarriers loaded with simvastatin were injected locally into the scar. Each mouse was injected only once in the murine ear keloid after successful modeling.

#### 2.3.2 Evaluation of the Scar Treatment Effect

The Vancouver scar scale (VSS) [14] was used to assess the efficacy of scar treatment. A score of 0 is considered normal, and a lower score indicates better efficacy in treating the scar.

#### 2.3.3 Hematoxylin and Eosin Staining

At the end of the treatment in eight mice, the heart, liver, and kidney were stained with hematoxylin and eosin (HE) and observed under a light microscope, and some damage was caused to the myocardium in the Simvastatin group, with some of the myofibers disorganized and broken, and the normal anatomical structure was lost, and in the liver, the confluent area was infiltrated by localized inflammatory cells in the Simvastatin group, and normal structure was lost, and the same result was observed in the kidney. In the kidney, the normal glomerular structure of the kidney disappeared. In the mice treated with CNDS@Simvastatin, there was no obvious macromolecule deposition in the heart, liver, and kidney, and no obvious disruption of the tissue structure, which was not significantly different from that of the control group.

### 2.4 Statistical Analysis

All experiments were repeated three times or more. Statistical analysis and plotting were done using SPSS 26.0, GraphPad Prism 9.5, and Adobe Illustrator 2021 software. The *t*‐test was used to compare between the two groups. A one‐way analysis of variance (ANOVA) was used for statistical analysis among multiple groups. The chi‐square test was used for enumeration data. The measurement data were expressed as the mean ± standard deviation. A *p* value of < 0.05 was considered statistically significant (*p < 0.05; **p < 0.01; ***p < 0.001).

## 3 Results

### 3.1 Morphology of CNDS@Simvastatin

**FIGURE 2:**
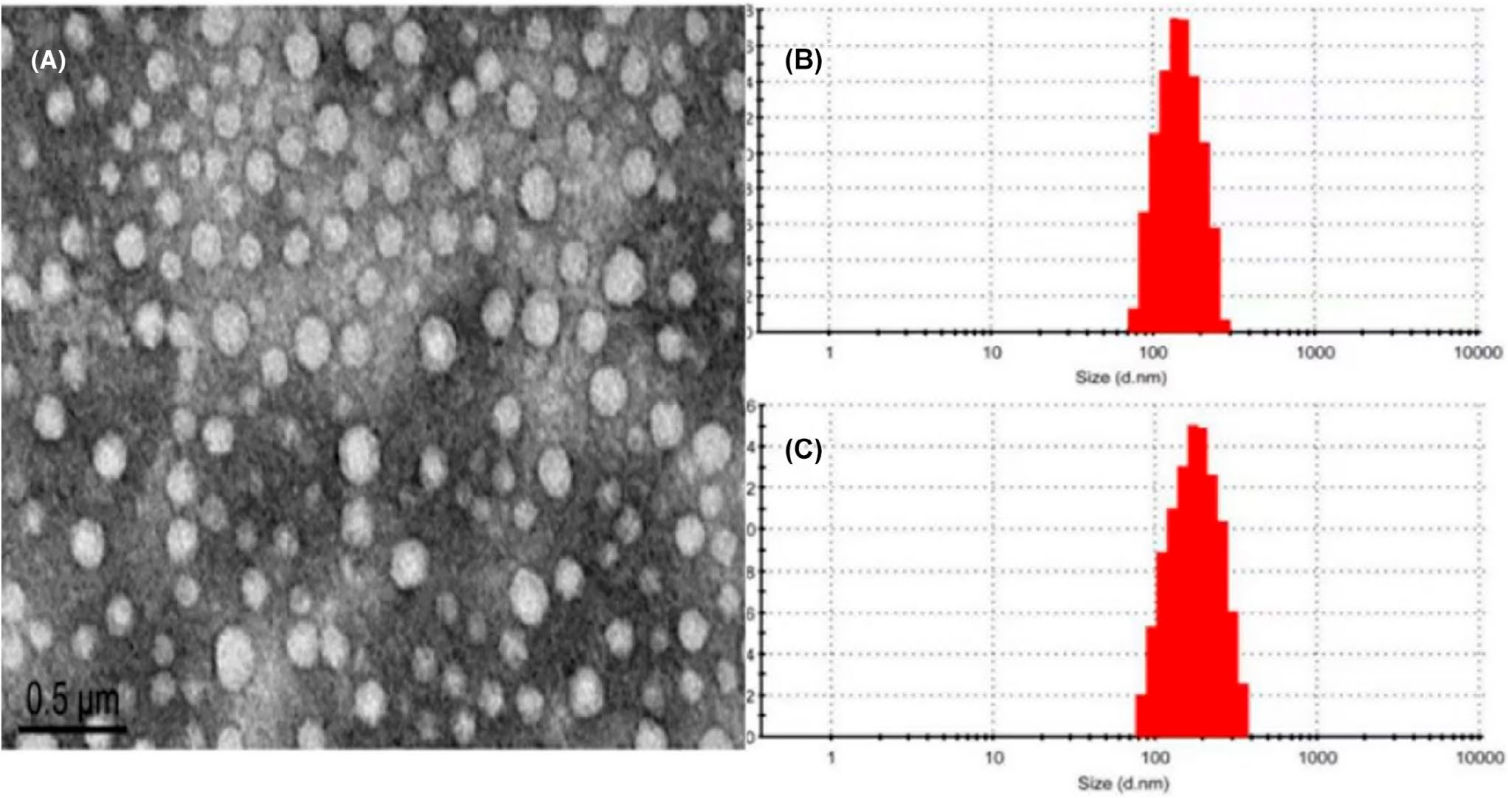
(A) Transmission electron microscopy of CNDS@Simvastatin; (B) Particle size distribution of CNDS; (C) Particle size distribution of CNDS@Simvastatin.

Following the successful preparation of PEG‐PLGA‐Hyd‐loaded simvastatin, that is, CNDS‐Simvastatin, the in vivo and in vitro characterizations of CNDS@ Simvastatin were assessed. CNDS@Simvastatin exhibited a uniformly dispersed spherical shape with no adhesions between particles and a favorable morphology, as shown in Figure 2A.

### 3.2 Particle Size and Zeta Potential of CNDS and CNDS@Simvastatin

As seen in Figure 2B,C, the particle sizes of CNDS and CNDS@Simvastatin were relatively homogeneous and normally distributed, and the distribution range was narrow. The particle size of CNDS measured 151 nm, with a Zeta potential of −15.32 mV and a dispersion (polydispersity index, or PDI) of 0.214. Upon loading, the CNDS@Simvastatin particle size increased to 178 nm, with a Zeta potential of −13.60 mV and a dispersion (PDI) of 0.228.

### 3.3 Efficiency and Drug Loading Capacity of CNDS@Simvastatin Encapsulation

The entrapment efficiency and drug loading of CNDS@Simvastatin were 67.44% and 3.27%, respectively. These results indicate that CNDS@Simvastatin had good entrapment efficiency and drug loading capacity, fulfilling the requirements of nanomedicine carriers.

### 3.4 Behavior of Simvastatin Sensitive Release by CNDS@Simvastatin Acid

**FIGURE 3:**
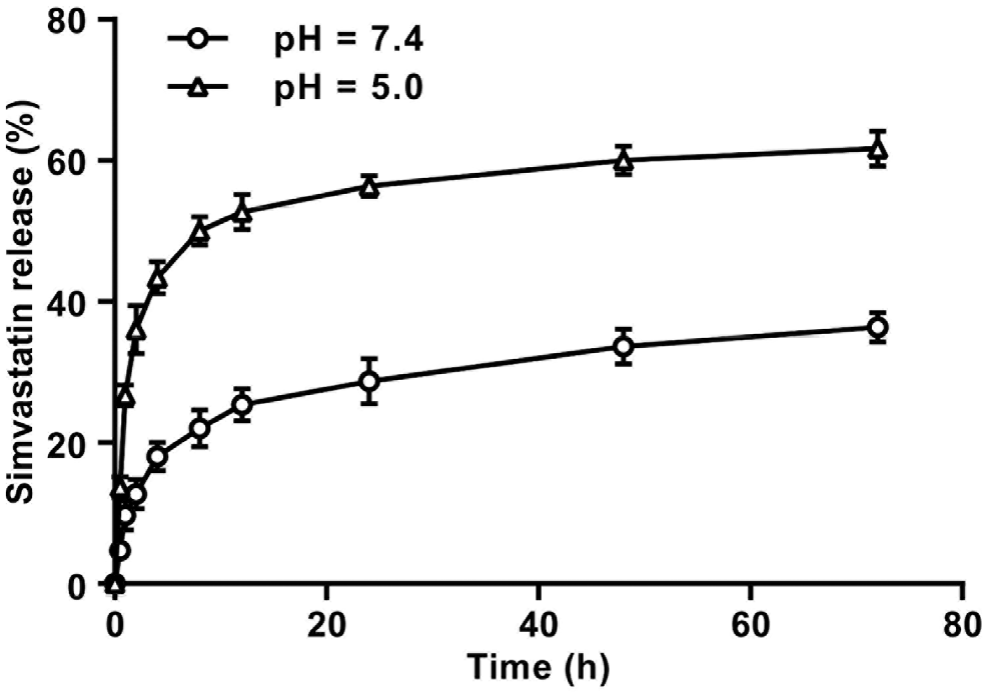
Drug release curves of simvastatin released from CNDS@Simvastatin in different pH solutions.

CNDS@Simvastatin exhibited slow release characteristics in both acidic and alkaline solutions, demonstrating sustained release behavior. Notably, drug release was greater at pH 5.0, with 61.67% cumulative release at 72 h, which was significantly higher than the 36.33% release at pH 7.4 (Figure 3). These findings highlight the pH‐sensitive nature of CNDS@Simvastatin, with enhanced drug release in acidic environments than in alkaline environments.

### 3.5 Stability Evaluation of CNDS@Simvastatin

CNDS@Simvastatin was found to be stable, showing resistance to phagocytosis by macrophages after release into the bloodstream and efficiently entering target cells (Figure S1).

### 3.6 Evaluation of Cytotoxicity of CNDS@Simvastatin

**FIGURE 4:**
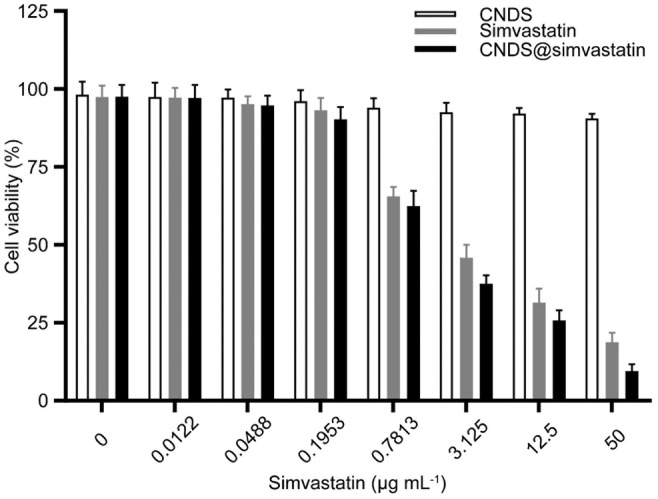
Cytotoxicity test of each group.

CNDS was essentially noncytotoxic, while simvastatin and CNDS@Simvastatin gradually decreased the activity of human umbilical vein endothelial cells with increasing simvastatin concentrations. CNDS@Simvastatin demonstrated greater inhibition of cell viability compared to simvastatin alone (Figure 4). These results suggest that the copolymer acid‐sensitive nanomedicine carriers are nontoxic and have favorable biosafety. CNDS@Simvastatin could inhibit the activity of cells, and its efficacy was better than that of simvastatin alone.

### 3.7 Evaluation of Cellular Uptake of CNDS@Simvastatin

Both CNDS and CNDS@Simvastatin were observed to be taken up by cells, and there was not much difference between the two (Figure S2).

### 3.8 Immunofluorescence Staining Using the Proliferating Cell Nuclear Antigen Antibody and the Cell Growth and Proliferation in Each Group at Different Time Points as Observed by Confocal Microscopy

**FIGURE 5:**
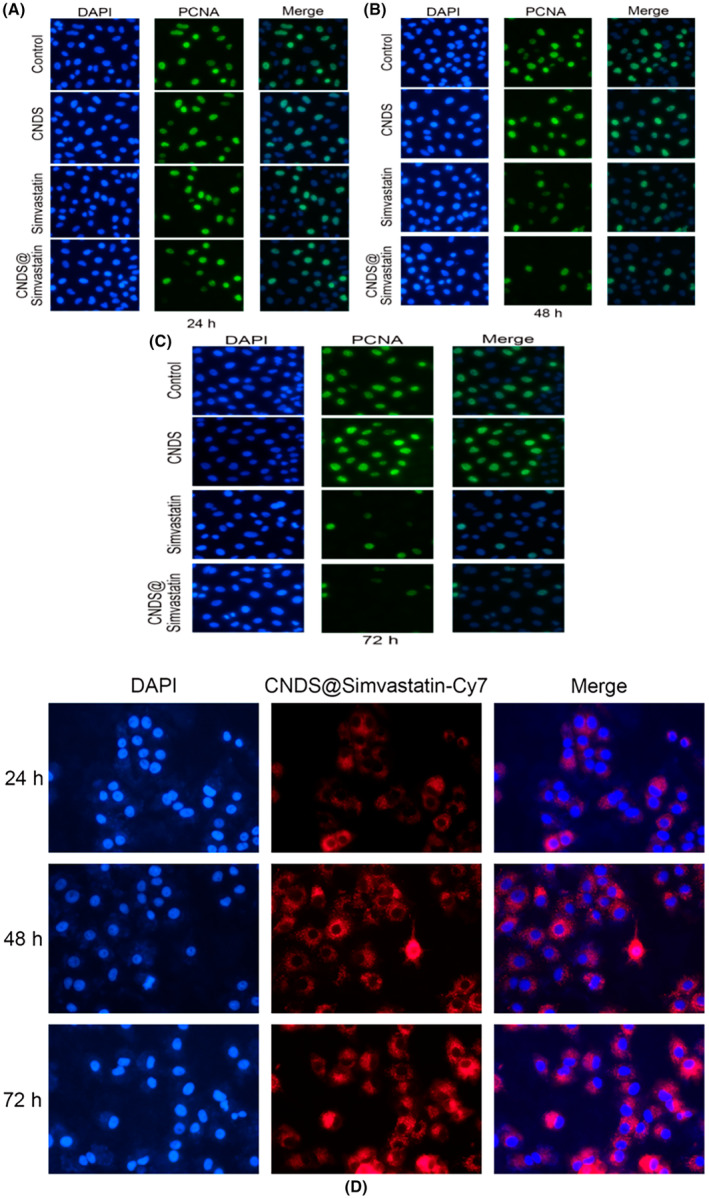
(A–C) Immunofluorescence staining using proliferating cell nuclear antigen (PCNA) antibody to observe the cell growth and proliferation of each group performed at 24 h (A); 48 h (B); and 72 h (C) using confocal microscopy; and (D) Drug release after CNDS@Simvastatin entered fibroblasts at 24, 48, and 72 h.

As shown in Figure 5A–C, proliferating cell nuclear antigen (PCNA) protein was mainly expressed in the nucleus, as revealed by the results of confocal microscopy. There was no significant difference between the control group and the CNDS group after the addition of green fluorescent PCNA protein to fibroblasts treated with each group of drugs. However, after treatment of cells with simvastatin, the PCNA protein expression levels in fibroblasts decreased progressively and more significantly over time, with the inhibitory effect more pronounced in the CNDS@Simvastatin group. These findings highlight several key points: (1) The CNDS group demonstrated no toxicity or lethality to fibroblasts and had good biosafety. (2) The simvastatin group could inhibit the proliferation of fibroblasts, with CNDS@Simvastatin demonstrating better efficacy in inhibiting the proliferation of fibroblasts than the simvastatin group. (3) Both simvastatin and CNDS@Simvastatin showed a gradual inhibition of fibroblast proliferation over 72 h, exerting their effects within the nuclei of cells.

### 3.9 Slow Release of CNDS@Simvastatin Into Fibroblasts

The DAPI that was used to stain the nucleus emitted blue fluorescence, and the CNDS@Simvastatin nanospheres were labeled with red Cy7 fluorescence, as shown in Figure 5D. Confocal microscopy revealed that at 24 h after entering the fibroblasts, CNDS@Simvastatin did not overlap between blue and red fluorescence; however, at 48 h and 72 h, the complete overlap between the blue and red fluorescence signals was observed, reaching the peak. This indicates that the CNDS@Simvastatin nanospheres could initiate sustained drug release around the nucleus between 48 and 72 h after entering the fibroblasts.

### 3.10 Q‐PCR Results in Detecting the Inhibition of Col I and CTGF Expression in Fibroblasts at 24, 48, and 72 h in Each Group

**FIGURE 6:**
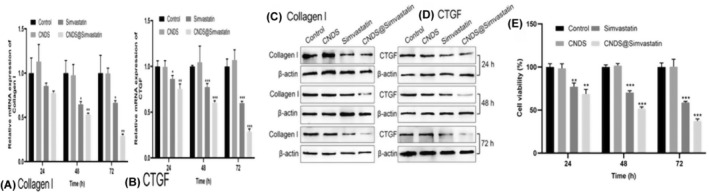
Collagen I and CTGF mRNA, protein, and fibroblast viability expression levels detected using Q‐PCR, Western blot, and CCK8 at 24, 48, and 72 h in the four groups. (A) MRNA expression level of Collagen I; (B) MRNA expression level of CTGF; (C) Collagen I protein expression level; (D) CTGF protein expression level; (E) Fibroblast viability expression level. *p < 0.05, **p < 0.01, ***p < 0.001.

From the details in Figure 6A, B, it can be seen that: (1) Treatment with simvastatin inhibited the mRNA expression levels of both Col Ι and CTGF, with the optimal treatment time point observed to be 72 h. (2) Compared with the simvastatin group, the mRNA expression levels of Col Ι and CTGF were significantly inhibited in the CNDS@Simvastatin group, and the difference was statistically significant (*p* < 0.001). (3) The simvastatin group could inhibit Col Ι and CTGF within 24 and 48 h, while the expression of Col Ι was lessened at 72 h. On the other hand, the CNDS@Simvastatin group exhibited a gradual and sustained release of simvastatin from 24 to 72 h, resulting in continuous inhibition of Col Ι and CTGF expression. The difference was statistically significant (*p* < 0.01).

### 3.11 Western Blot Results in Detecting the Inhibition of Col Ι and CTGF Expression in Fibroblasts in Each Group at 24, 48, and 72 h

As shown in Figure 6C,D, Western blot analysis revealed that (1) Simvastatin was effective in inhibiting the protein expression levels of Col Ι and CTGF, with the most pronounced inhibition observed at 72 h after initiation of treatment. (2) The inhibition of protein expression levels of Col Ι and CTGF was more significant in the CNDS@Simvastatin group than in the simvastatin group. (3) Compared with the simvastatin group, the CNDS@Simvastatin group demonstrated a sustained release of the drug over the 72‐h period, resulting in a continuous and uniform suppression of Col Ι and CTGF expression.

### 3.12 CCK8 Results Pertaining to the Expression Level of Fibrocyte Viability in Each Composition at Varying Time Points

A comparison of the four groups at varying time points, specifically 24, 48, and 72 h (Figure 6E), reveals that both the CNDS@Simvastatin and simvastatin groups were able to inhibit cell viability, and the cell viability inhibition was the most significant at 72 h from the treatment initiation (*p* < 0.01). Compared with the simvastatin group, the release of simvastatin in the CNDS@Simvastatin group was more gradual and uniform over a 72‐h duration, and this sustained mechanism resulted in a continuous inhibition of fibroblast viability (*p* < 0.001).

### 3.13 Detection of the Apoptosis Level in Fibrocytes of Each Group Using Flow Cytometry

**FIGURE 7:**
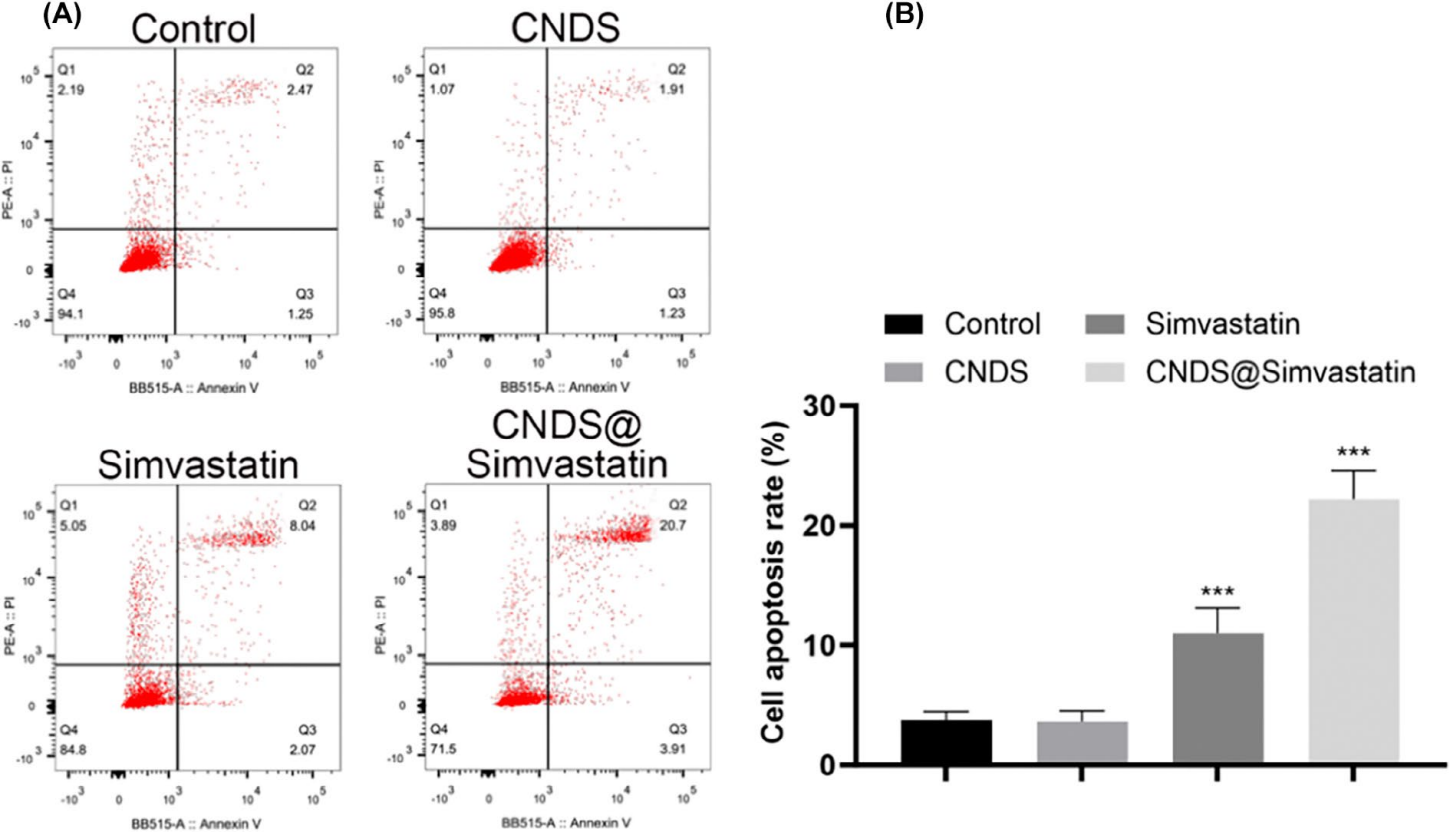
(A) Expression levels of apoptosis in fibrocytes of the four groups detected using flow cytometry. (B) Statistical data of apoptosis detected using flow cytometry. *p < 0.05, **p < 0.01, ***p < 0.001.

All four groups elicited heightened expression of fibroblast apoptosis, with CNDS@Simvastatin inducing the highest expression level of fibroblast apoptosis. Notably, CNDS@Simvastatin had a significantly better effect than the simvastatin group, the CNDS group, and the control group (*p* < 0.001), as shown in Figure 7.

### 3.14 Clinical Observations of Keloid Treatment Efficacy in the Mice Ears of Each Group

**FIGURE 8:**
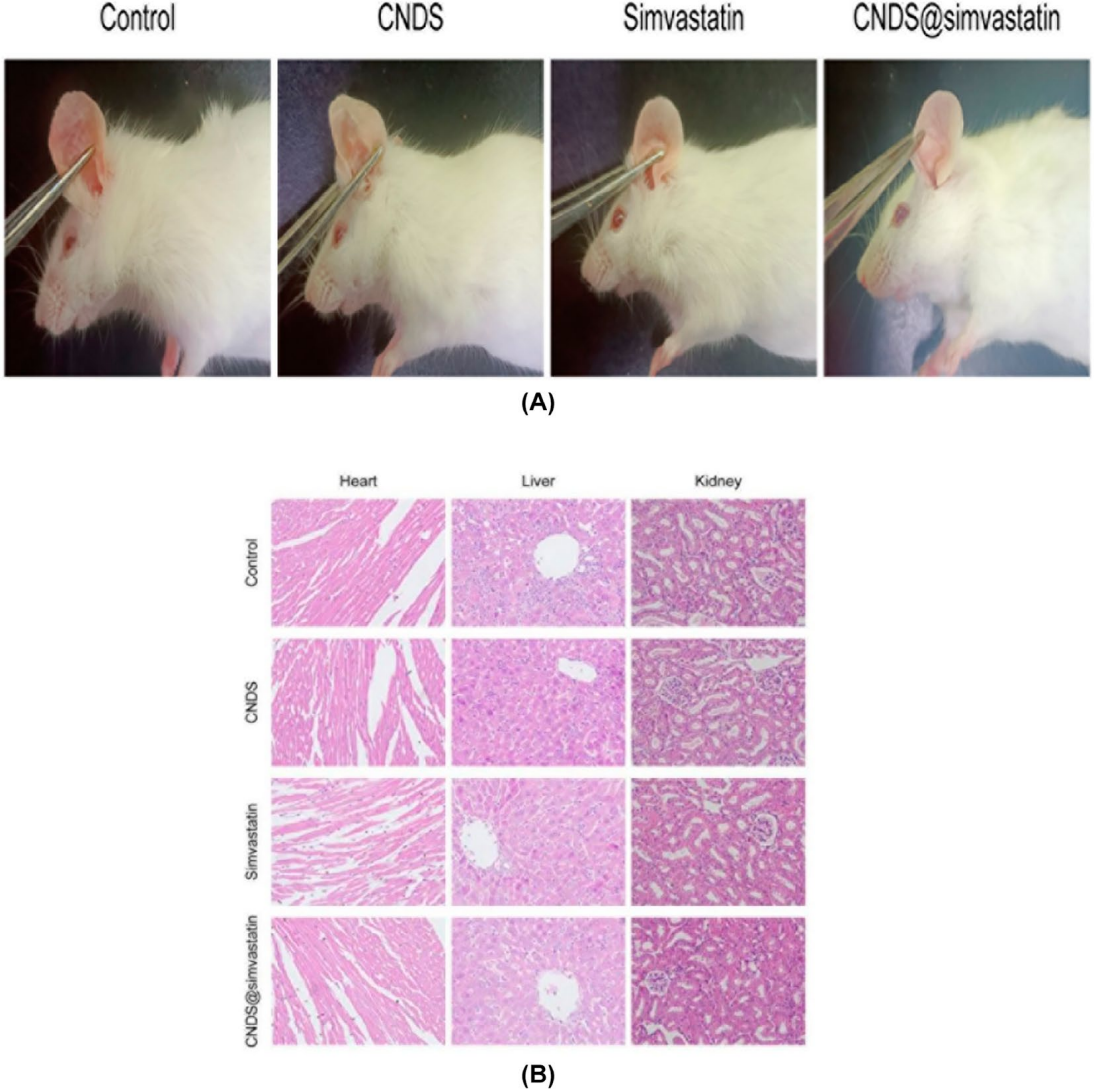
(A) Therapeutic evaluation of keloid animal models in the mice ears of each group; (B) HE staining of heart, liver, and kidney organs in mice after keloid treatment in the ears of each group.

The results showing the efficacy of keloid treatment in mice ears across all four groups are detailed in Figure 8A. Initially, the observations in the four groups based on the scar measuring scale were as follows: In the CNDS group, compared with the control group, there was no significant improvement effect, but there was no aggravation of the keloid condition in mice ears. In contrast, compared with the control group, the simvastatin group demonstrated notable improvements in the treated keloids in mice ears. This was evidenced by a significant reduction in the keloid area, as well as decreased length and thickness of the keloid, accompanied by lighter skin lesion color and softer texture. When compared with the simvastatin group, the CNDS@Simvastatin group had better therapeutic efficacy on the keloids. Improvements were noted in keloid length, width, height, color, and texture, with no obvious skin lesions detected on inspection and palpation.

### 3.15 Evaluation of the Efficacy on Keloids in the Mice Ears of Each Group Using VSS

As shown in Table 1, both the simvastatin and the CNDS@Simvastatin groups showed lower scores in the thickness, vascularity, pigmentation, and pliability of keloids compared to the control and CNDS groups (*p* < 0.05), indicating that simvastatin and CNDS@Simvastatin have therapeutic significance in treating keloids, and this was consistent with the clinical observations that were made. The CNDS@Simvastatin group had the lowest scores across all parameters as well as the lowest total scores (*p* < 0.05), indicating the best therapeutic effect on keloids in mice ears among the experimental groups.

**TABLE 1:** Vancouver scar scale (VSS) scores of keloids in the ears of mice in each group (X ± S score).

Groups	Thickness	Vascularity	Pigmentation	Pliability	Overall score
Control group	2 ± 0.38	1.4 ± 0.26	2 ± 0.10	4.2 ± 0.18	13.2 ± 0.30
CNDS group	2 ± 0.35	1.4 ± 0.24	2 ± 0.08	4.1 ± 0.16	11.6 ± 0.28
Simvastatin group	2 ± 0.30	1.4 ± 020	2 ± 0.08	3.2 ± 0.12	7.4 ± 0.25
CNDS@Simvastatin group	1 ± 0.26	1.2 ± 0.18	1 ± 0.16	1.8 ± 0.12	2.3 ± 0.20

### 3.16 RT‐PCR Results in Inhibition of Keloid Inflammatory Cytokines by CNDS@Simvastatin at Varying Time Points

RT‐PCR was used to detect the expression levels of keloid inflammatory cytokines, specifically interleukin‐1β (IL‐1β), interleukin‐6 (IL‐6), and tumor necrosis factor‐alpha (TNF‐α), inhibited by CNDS@Simvastatin on days 3, 7, 14, and 21. The CNDS@Simvastatin group exerted a gradual and continuous inhibition of keloid inflammation over the course of 21 days. Notably, the inhibited concentrations of inflammatory cytokines were ranked as IL‐6 > IL‐1β > TNF‐α (Figure S3).

### 3.17 Biocompatibility Evaluation of CNDS@Simvastatin

As shown in Figure 8B, in the Simvastatin group, it was observed that there was some damage to the myocardium, characterized by disorganized and broken muscle fibers, resulting in the loss of the original anatomical structure. In addition, local inflammatory cell infiltration in the portal area and loss of normal structure were observed in the liver and in the kidney of the Simvastatin group; the normal glomerular structure of the kidney was compromised. However, no obvious macromolecular deposition was observed in the heart, liver, or kidney of mice treated with CNDS@Simvastatin, and there were no obvious tissue structural abnormalities, indicating compatibility comparable to the control group.

## 4 Discussion

Simvastatin, characterized by its lower water solubility and a short half‐life of 1–5 h, necessitates multiple intralesional injections [15, 16]. This poses challenges in clinical treatment as repeated injections not only cause significant discomfort to patients but also result in hard and thick scars that are difficult for medical staff to manage [1].

To address this issue, there has been a growing focus on the use of nanopolymer microspheres as drug carriers with low water solubility. PEG, one of the most widely used polymer carriers, is favored due to its nontoxic and nonirritating nature, as well as its excellent water solubility and lubrication properties [17]. Incorporating PEG can prolong the circulation time of the drug in the bloodstream and enhance the water solubility of simvastatin.

Another carrier offering promise is PLGA, a novel degradable hydrophobic organic polymer compound formed by random polymerization of lactic acid and glycolic acid [18]. With improved compatibility, nontoxicity, and efficient encapsulation, it is currently FDA‐certified as a leading polymer carrier, and it facilitates sustained release of the drug due to its hydrophobic nature [11, 12]. This controlled release mechanism prevents the lysis of striated muscle caused by repeated intralesional injections of simvastatin and the cellular toxicity due to cumulative doses while reducing the burden on medical personnel staff to inject keloids multiple times.

The PEG‐modified PLGA, forming the PEG‐PLGA copolymer carrier, demonstrates several favorable characteristics, including good blood stability, sustained release capability, biocompatibility, and renal excretion without causing aggregation or deposition within the human body. In our experiment, Hyd, a pH‐sensitive chemical bond, was added to PEG‐PLGA nanomicelle microspheres to form CNDS. Simultaneously, simvastatin was co‐encapsulated to create CNDS@Simvastatin to trigger Hyd dissociation in the acidic environment of lysosomes within fibroblasts. This resulted in the slow release of simvastatin mediated by PLGA, which then inhibited CTGF, Col Ι, and fibroblast activity. However, there is no clinical data available on the effectiveness of CNDS@Simvastatin in keloid treatment.

In this study, PEG‐PLGA‐Hyd Simvastatin‐loaded nanospheres, named CNDS@Simvastatin, were developed, and their properties and efficacy were tested in vivo. First, the morphology of CNDS@Simvastatin was observed using TEM, which revealed uniformly dispersed spherical structures without adhesion between them and good morphology. Subsequently, DLS findings demonstrated that the nanosystem particle size, zeta potential, encapsulation efficiency, and drug loading fulfilled the requirements of nanodrug carriers.

Additionally, to investigate the drug release behavior of CNDS@Simvastatin in fibroblasts under the acidic conditions of lysosomes, CNDS@Simvastatin was exposed to pH 7.4 PBS and pH 5.0 PBS solutions. We found that CNDS@Simvastatin exhibited a gradual release profile in both acidic and alkaline solutions, with significantly higher drug release at pH 5.0 compared to pH 7.4. This highlights the pH‐sensitive characteristics of CNDS@Simvastatin, with a greater amount of simvastatin released in acidic environments than in alkaline ones. This outcome aligns with the intended design objective of developing CNDS@Simvastatin.

Next, the effects of CNDS, simvastatin, and CNDS@Simvastatin on human cytotoxicity were compared using the MTT assay. CNDS exhibited good biosafety without inducing toxicity; both simvastatin and CNDS@Simvastatin showed a dose‐dependent decrease in cell viability; and CNDS@Simvastatin inhibited cell viability better than simvastatin alone.

To further answer whether CNDS@Simvastatin could be effectively released in fibroblasts and to determine the timing and site of drug release, in vitro experiments were performed. We found that CNDS@Simvastatin accumulated around the nucleus between 48 and 72 h post‐administration, indicating the commencement of drug release. The slow release of simvastatin led to a continuous and uniform inhibition of the expression of CTGF and Col Ι in fibroblasts over 72 h, and this sustained release in turn contributed to the gradual suppression of keloid fibroblasts and collagen fibers.

Lastly, the clinical efficacy of CNDS@Simvastatin in the treatment of keloids was tested through in vivo animal experiments. The expression of TNF‐α, IL‐1β, and IL‐6—key inflammatory cytokines involved in keloid formation—was measured, and the biocompatibility of CNDS@Simvastatin was evaluated to ascertain its impact on important organs. Our results indicated that CNDS@Simvastatin was the most effective in the treatment of keloids, sustaining the inhibition of keloid inflammation over a 21‐day period. Notably, CNDS@Simvastatin demonstrated no adverse impact on important organs in mice, indicating its lack of toxicity and high biosafety.

However, growth factors that play an important role in stimulating fibroblasts and inducing collagen synthesis include TGF‐β, FGF, and VEGF. We were unable to evaluate their gene expression profiles, which is a limitation of this study.

## 5 Conclusion

In this study, a novel injectable simvastatin‐loaded copolymer acid‐sensitive nanocarrier, CNDS@Simvastatin copolymer nanospheres, was developed to treat keloids. CNDS@Simvastatin demonstrated good physicochemical properties as well as favorable biocompatibility. Incorporating acid‐sensitive groups, the drug was found to have an enhanced ability to penetrate the acidic environment of cells, which improved the drug utilization rate. Additionally, the prolonged administration of simvastatin enhanced drug osmosis and retention. The CNDS@Simvastatin copolymer nanospheres, characterized by high efficiency, sustained release, and nontoxicity, represent an ideal and innovative drug for the treatment of keloids, with significant potential for clinical application.

## Ethics Statement

All experiments were evaluated and approved by the Ethics Committee of Fourth Affiliated Hospital of Harbin Medical University (2022‐DWSYLLCZ‐94). Principles of Laboratory Animal Care’ (NIH Publication Vol 25, No. 28 revised 1996; http://grants.nih.gov/grants/guide/notice‐files/not96‐208.html) were followed, as well as specific national laws (e.g., the current version of the German Law on the Protection of Animals) where applicable.

## 6 Conflicts of Interest

The authors declare no conflicts of interest.

## Supporting information


**Figure S1.** Stability evaluation of CNDS and CNDS@Simvastatin in normal saline solution. The average particle size of CNDS is about 150 nm, and the average particle size of CNDS@Simvastatin is about 180 nm.

**Figure S2.** Evaluation of CNDS and CNDS@Simvastatin uptake in human umbilical vein endothelial cells. A: CNDS uptake after entering human umbilical vein endothelial cells. B: CNDS@Simvastatin uptake into human umbilical vein endothelial cells. After CNDS and CNDS@Simvastatin entered the cells, the blue and green fluorescence completely overlap.

**Figure S3.** RT‐PCR used to determine the expression of inhibiting keloid inflammation in the control group and CNDS@Simvastatin group on days 3 (A), 7 (B), 14 (C), and 21 (D), respectively.

## Data Availability

The data that support the findings of this study are available from the corresponding author upon reasonable request.
